# Construction and regulation of microbial cell factories for enhancing the biosynthesis of O-acetyl-l-homoserine in *Escherichia coli* W3110

**DOI:** 10.1042/BCJ20243022

**Published:** 2026-02-02

**Authors:** Kun Niu, Yi-Fan Zhao, Zi-Xuan Zhang, Yao-Yao Wang, Kai-Di Xiang, Sen Cui, Zhi-Qiang Liu, Yu-Guo Zheng

**Affiliations:** 1 State Key Laboratory of Green Chemical Synthesis and Conversion, Zhejiang University of Technology, Hangzhou, P. R., 310014, China; 2 Zhejiang Key Laboratory of Bioorganic Synthesis, College of Biotechnology and Bioengineering, Zhejiang University of Technology, Hangzhou, P. R., 310014, China

**Keywords:** glucose transport system, NOG pathway, O-acetyl-L-homoserine, regulation of cell division

## Abstract

O-Acetyl-l-homoserine (OAH) is a versatile platform compound with extensive potential applications. It is a key precursor for the synthesis of l-methionine and S-adenosylmethionine. Currently, the microbial fermentation process for the production of OAH still faces challenges, such as low fermentation yield and long fermentation period. In this study, the supply of key precursors, including l-aspartic acid, l-homoserine, and acetyl-CoA, was firstly enhanced, which increased the OAH production from 7.25 g/l to 12.95 g/l in shaking flask fermentation. Subsequently, the non-oxidative glycolysis pathway (NOG pathway) was constructed and optimized to minimize the carbon loss and improve the carbon sources utilization, resulting in an increase in OAH production to 15.59 g/l. Finally, by accelerating cell division and enhancing the glucose transport system, OAH production was further improved to 17.23 g/l. The OAH production of the engineered strain OAH23 achieved a production level of 66.25 g/l in a 5 l bioreactor for 68 h, with the yield of 0.41 g/g glucose. The metabolic regulation strategy outlined in this study offers valuable insights for the efficient biosynthesis of OAH and other acetylated amino acids in *E. coli*.

## Introduction

O-Acetyl-l-homoserine (OAH) is an acylated amino acid, which can be hydrolyzed to l-homoserine, and further synthesized to homoserine lactone, γ-butyrolactone [1], 1, 4-butanediol [2], and the herbicide glyphosate [3]. In addition, OAH serves as a crucial precursor for the biosynthesis of l-methionine. As an essential amino acid for humans, l-methionine is widely used in various fields, including the feed industry, food industry [4], pharmaceuticals industry [5], and cosmetics industry, with a global annual demand exceeding 1.6 million tons [6]. OAH can react with methanethiol to generate l-methionine by acetylhomoserine sulfhydrylase (MetY), and this synthetic route has been used in the industrial production of l-methionine [7]; therefore, with the growing demand for glyphosate and l-methionine, the biosynthesis of OAH with microbial cell factories has attracted increasing attention from researchers.

**Figure 1:**
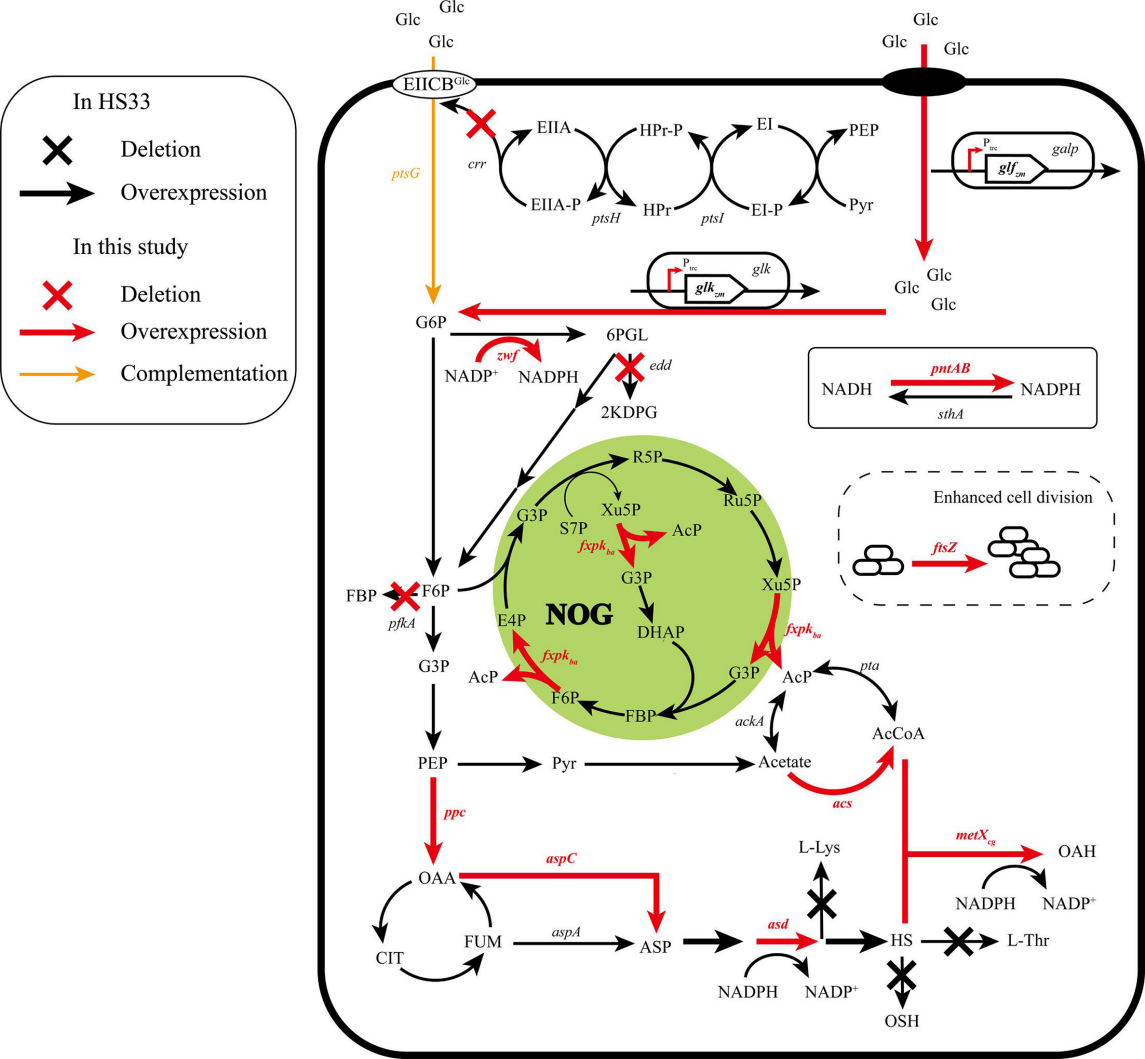
Metabolic pathway of the OAH-producing strain. Glc, glucose; G6P, glucose-6-phosphate; F6P, fructose-6-phosphate; G3P, glyceraldehyde-3-phosphate; 6 PGL, 6-phospho-d-gluconate; PEP, phosphoenolpyruvate; Pyr, pyruvate; AcP, acetyl phosphate; AcCoA, acetyl-CoA; CIT, citrate; OAA, oxaloacetic acid, FUM, fumaric acid ; ASP, aspartic acid ; ASP-P, aspartyl-phosphate; ASP-SA, aspartate semialdehyde; HS, l-homoserine; l-Lys, l-lysine; l-Met, l-methionine; l-Thr, l-threonine; OSH, O-succinyl-l-homoserine.

*Escherichia coli* (*E. coli*), characterized by its well-defined genetic background, complete carrier receptor system, rapid growth, and simple cultivation condition, has been utilized for the production of l-aspartate family amino acids, such as l-asparagine, l-threonine, and l-lysine [8,9], and showed great potential in the production of OAH [10]. The biosynthetic pathway of OAH in *E. coli* from glucose can be divided into three modules (Figure 1), glucose transport module, l-aspartic acid and l-homoserine biosynthesis module, and acetyl-CoA supply module. Glucose can be metabolized by the glycolysis pathway (EMP), pentose phosphate pathway (PPP), and tricarboxylic acid cycle (TCA cycle) to synthesize the key precursor l-aspartic acid (ASP). The biosynthesis of l-aspartic acid can be achieved in two ways, one of which is catalyzed by aspartic aminotransferase (AspC, encoded by gene *aspC*) and the substrate is oxaloacetic acid (OAA). The other pathway is catalyzed by aspartate oxidase (AspA, encoded by gene *aspA*) with fumaric acid (FUM) as the substrate. l-Aspartic acid is further synthesized into l-homoserine and then reacts with acetyl-CoA under the catalysis of homoserine acetyltransferase (MetX, encoded by gene *metX*) to produce O-acetyl-l-homoserine [11]. However, *E. coli* inherently lacks the essential enzyme MetX, necessitating the introduction of an exogenous MetX. Currently, a variety of MetX enzymes derived from diverse bacterial strains have been reported to efficiently catalyze the acetylation of l-homoserine, including those from *Corynebacterium glutamicum* [12], *Leptospira spp*. [13], *Pseudomonas aeruginosa, and Mycobacterium pilotrichum* [14].

There are some literatures reporting on OAH biosynthesis. Kase *et al*. obtained a mutant strain of *C. glutamicum*, which was resistant to l-methionine analogs and was used to produce OAH with an accumulation of 10.5 g/l [15]. Li *et al*. investigated the production of OAH with *C. glutamicum* and enhanced the production to 17.4 g/l by overexpression of l-homoserine dehydrogenase and MetX, then successfully identified the limiting factors and improved the OAH titer to 25.9 ‍g/l ‍ [16,17]. Liu *et al.* reported the OAH production by *E. coli* using glycerol. The glycerol uptake system and the glycerol oxidation pathway were modified, and the gene *metX* from *C. glutamicum* was introduced into the engineered strain. The results indicated that 9.42 g/l OAH accumulated in shake flask fermentation with pure glycerol [18]. Wei *et al*. used *E. coli* W3110 as the starting strain and constructed the OAH biosynthesis pathway by protein engineering and metabolic engineering, which enhanced the OAH production to 62.7 g/l [10].

Based on the previous studies, the metabolic pathways of OAH biosynthesis were analyzed and modified in this study. It is speculated that the biosynthesis of OAH may be constrained by the following factors, such as inadequate supply of precursors, low utilization efficiency of carbon sources, and compromised bacterial growth viability. To address these limitations, the present study adopted a suite of targeted strategies to promote OAH biosynthesis. First, the metabolic fluxes of the precursor substances, including l-aspartic acid, l-homoserine, and acetyl-CoA, were enhanced to alleviate precursor shortage. Second, the nonoxidative glycolysis (NOG) pathway was established to effectively reduce carbon loss and improve carbon source utilization efficiency. Third, the glucose transport system was optimized and cell division was regulated to enhance glucose utilization and shorten the fermentation cycle. Finally, a highly efficient OAH producing strain was obtained. When cultured in a 5 l bioreactor, this strain achieved an OAH yield of 66.25 g/l. The metabolic regulation strategy proposed in the present paper provides a reference value for the efficient biosynthesis of OAH and other acetylated amino acids in *E. coli*.

## Results

### Strengthening the supply of the key precursors l-aspartic acid and l-homoserine

**Figure 2:**
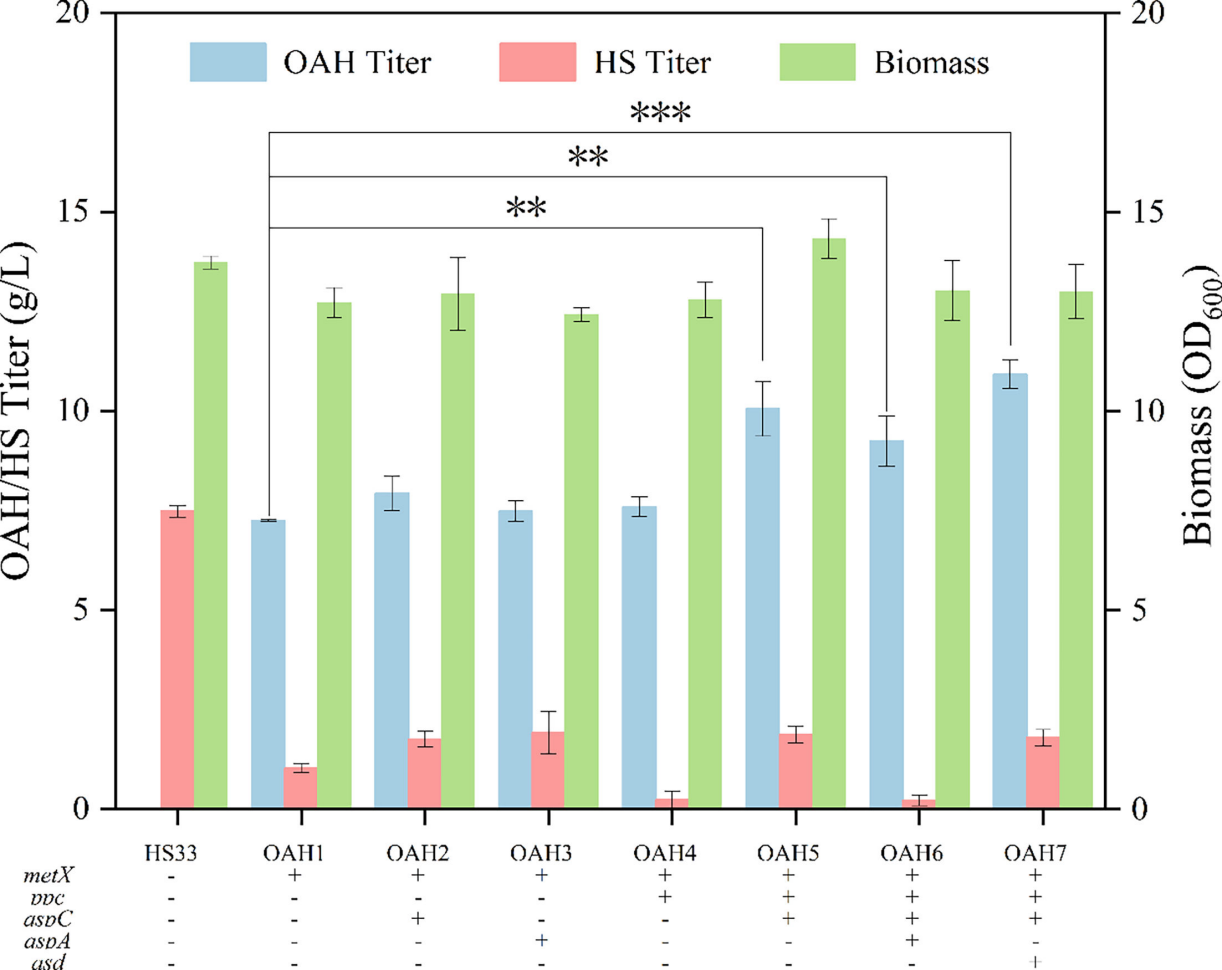
Effect of strengthening the supply of l-aspartic acid and l-homoserine on OAH production.

In the previous study, an l-homoserine-producing strain HS33 was constructed in our lab [19]. Then the plasmid carrying the gene *metX_cg_* was introduced into the genome of strain HS33 to obtain the OAH synthesis chassis strain OAH1. The results in Figure 2 showed that strain OAH1 had the OAH production ability of 7.25 g/l in the shaking flask.

l-Aspartic acid is a key intermediate metabolite for the biosynthesis of l-homoserine and its derivatives in *E. coli*, so enhancing the supply of l-aspartic acid may have an impact on OAH accumulation. It has been mentioned above that there are two l-aspartic acid synthesis pathways, AspC pathway and AspA pathway. Although both of the substrates, oxaloacetic acid and fumaric acid, are intermediate metabolites of the TCA cycle, the NADPH produced by the two pathways is different [20,21]. Therefore, in this study, the trc promoter was introduced to strain OAH1 to strengthen the AspC and AspA pathways, respectively, resulting in the engineered strains OAH2 and OAH3.

In addition, phosphoenolpyruvate carboxylase (encoded by the gene *ppc*) can catalyze phosphoenolpyruvate to oxaloacetic acid, a precursor of l-aspartic acid. Enhancing the expression of this enzyme may increase the supply of oxaloacetic acid and promote the synthesis of l-aspartic acid [22]. At the same time, the enzyme can reduce the loss of carbon sources by capturing one molecule of carbon dioxide [21,23]. Therefore, the strain OAH4 was constructed by strengthening the gene *ppc* with trc promoter. The results in Figure 2 showed that when the trc promoter was used to enhance the *aspC*, *aspA,* and *ppc* genes, the OAH yields increased by 9.51%, 3.31%, and 4.83%, respectively, indicating the enhancement of the biosynthesis metabolic flux of the precursor l-aspartic acid could improve the OAH accumulation. Subsequently, the genes of *aspC* and *aspA* were strengthened based on strain OAH4 by individually replacing their native promoters with the trc promoter, aiming to enhance the efficiency of their expression regulation, resulting in strains OAH5 and OAH6. Strain OAH5 showed higher OAH titer of 10.06 g/l.

The aspartic hemialdehyde dehydrogenase (encoded by the gene *asd*) was the key enzyme during the catalyzing process of l-aspartic acid to l-homoserine. Sun *et al*. increased the l-homoserine production by 28% by strengthening the gene *asd* [24]. In order to further increase the OAH production, the gene *asd* was strengthened by the trc promoter based on strain OAH5, and strain OAH7 was obtained. The OAH titer of strain OAH7 increased to 10.92 g/l (Figure 2), an increase of 50.62% compared with strain OAH1. The results indicated that the modification of the l-homoserine synthesis pathway was helpful for the production of OAH.

### Optimization of the carbon flow allocation to increase the supply of acetyl-CoA

**Figure 3:**
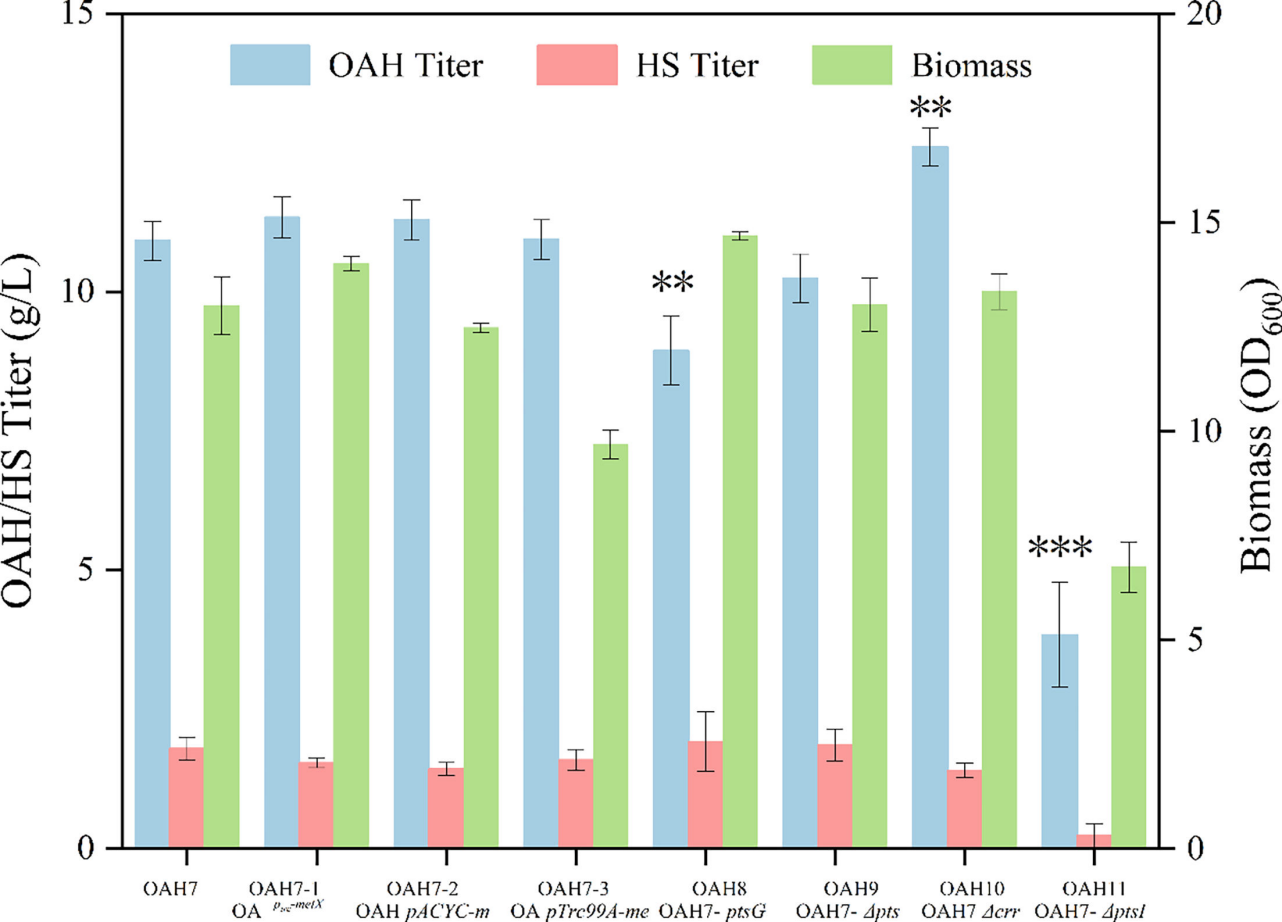
Effect of retrofitting PTS system on OAH production.

From the above results, it indicated that l-homoserine was accumulated in the broth, which indicates that the enzyme activity of MetX or the supply of another precursor acetyl-CoA might be insufficient during the OAH synthesis. Therefore, the gene *metX_cg_* was subsequently strengthened on the genome, pACYC plasmid, and pTrc99a plasmid based on strain OAH7, respectively. The results in Figure 3 indicated that strengthening the gene *metX_cg_* on the genome (strain OAH7-1) had an obvious promoting effect on OAH production, which could increase to 11.34 g/l. However, it indicated that there still existed about 1 g/l l-homoserine in the broth. Therefore, to improve the OAH production, the supply of another precursor acetyl-CoA was modified in the following studies.

Phosphoenolpyruvate (PEP) is one of the key precursors during acetyl-CoA synthesis in *E. coli*, and it also participates in a variety of metabolic reactions. PEP is directly involved in energy production reactions, such as substrate-level phosphorylation of ADP, or indirectly as a precursor of acetyl-CoA [25]. In *E. coli*, glucose is first transported into the cytoplasm through the PEP: carbohydrate phosphotransferase systems (PTS). One mole of PEP is required for transport and phosphorylation of one mole of glucose. When *E. coli* transports glucose, PEP connects the glycolytic pathway with the PTS transport system to form a phosphorylation loop, which consumes 50% of PEP to provide the necessary phosphorylation energy; therefore, it would increase the loss of carbon sources and greatly limit the supply of OAH precursors [26,27]. The proteins encoded by the genes of *ptsG*, *ptsH*, *crr,* and *ptsI* play different roles in glucose transport in the PTS system and are also involved in many functions, such as carbon metabolism, nitrogen source metabolism, cell membrane formation, and the adaptation of bacteria to different environments. Consequently, the knockout of various key genes results in distinct outcomes in the fermentation process [28]. In the chassis cell, the gene *ptsG* has been knocked out, thus the gene *ptsG* was firstly replenished on strain OAH7-1 to obtain strain OAH8, and then strains of OAH9, OAH10, and OAH11 were constructed by knocking out *ptsH*, *crr,* and *ptsI*, respectively, based on strain OAH8. The fermentation results in Figure 3 indicated that knocking out the genes of *ptsH* and *crr* could promote the OAH production to some extent, with the deletion of the gene *crr* (strain OAH10) having the most substantial impact on enhancing OAH production, which increased to 12.60 g/l, 11.11% higher than that of strain OAH7-1 [28,29]. This result may be attributed to the fact that the deletion of the gene *crr* does not severely impede glucose uptake, while simultaneously redirecting carbon flux towards PEP and subsequent precursors. However, the deletion of the gene *ptsI* resulted in a significant inhibition of bacterial growth and a substantial decrease in OAH fermentation yield to 3.84 g/l. Although previous studies have reported that knocking out the *ptsI* gene can enhance substrate utilization [30], our findings align with reports indicating that such knockout leads to a reduced glucose uptake rate and slower bacterial growth [31].

During the metabolic pathways, PEP was further metabolized to produce pyruvic acid (Pyr), then to acetyl-CoA and acetic acid. Meanwhile, acetic acid could be catalyzed to acetyl-CoA by acetyl-CoA synthetase (ACS, encoded by gene *acs*), while phosphate acetyltransferase (PTA, encoded by the gene *pta*) and acetate kinase (ACK, encoded by the gene *ackA*) reversibly catalyze the conversion of acetyl-CoA to acetic acid. The above pathways maintain the recycling of acetic acid and acetyl-CoA, which provides flexibility for acetyl-CoA synthesis and its level [32]. However, promoting the pathways at the same time will lead to an ineffective cycle between acetic acid and acetyl-CoA and greatly waste the ATP [32].

**Figure 4:**
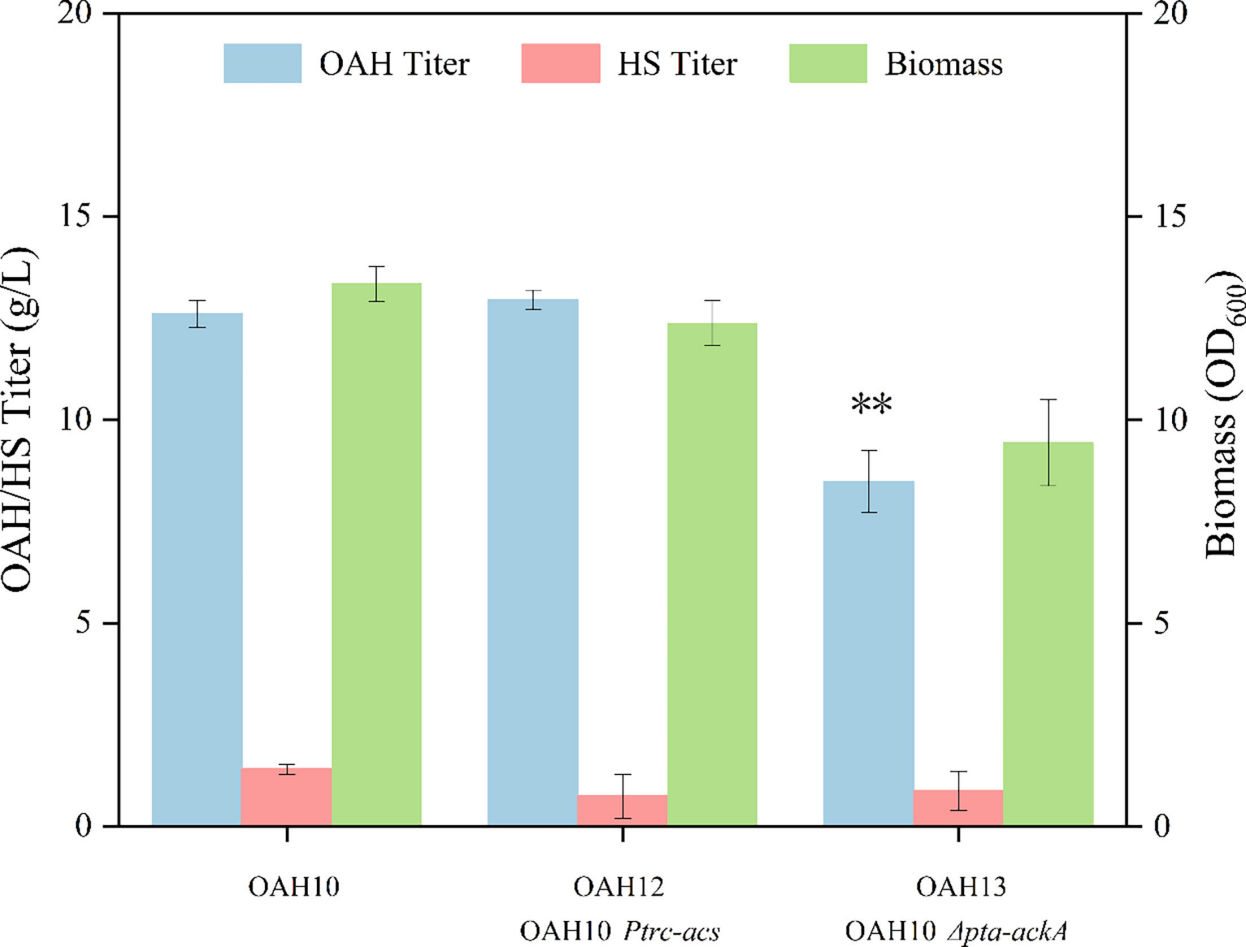
Effect of regulating the supply of acetyl-CoA on OAH production.

In order to explore the equilibrium mode of acetyl-CoA supply, the gene *acs* was strengthened by the trc promoter in strain OAH10 to obtain strain OAH12, and the gene *pta-ackA* was knocked out to construct strain OAH13. The shaking flask fermentation results of the two strains are shown in Figure 4. It indicated that the deletion of the gene *pta-ackA* significantly affected the cell growth, and the fermentation titer of OAH was only 8.48 g/l, much lower than that of strain OAH10. And the results in Supplementary Figure S1 showed that the deletion of the *pta-ackA* gene led to higher pyruvate accumulation, which may be the reason that affected the growth of bacteria and the supply of acetyl-CoA [33]. After overexpression of the gene *acs*, the fermentation titer of OAH was increased to 12.95 g/l, only 2.78% higher than that of strain OAH10. However, the l-homoserine accumulation was reduced from 1.41 g/l to 0.74 g/l, which indicated that the supply of acetyl-CoA had a certain promoting effect on the synthesis of OAH. Meanwhile, the result further illustrated the circulation between acetic acid and acetyl-CoA was affected by the culture environment, and the individual gene modification alone had little effect on the supply of acetyl-CoA and the accumulation of OAH.

### Enhancement of the carbon source utilization by constructing the NOG pathway

**Figure 5:**
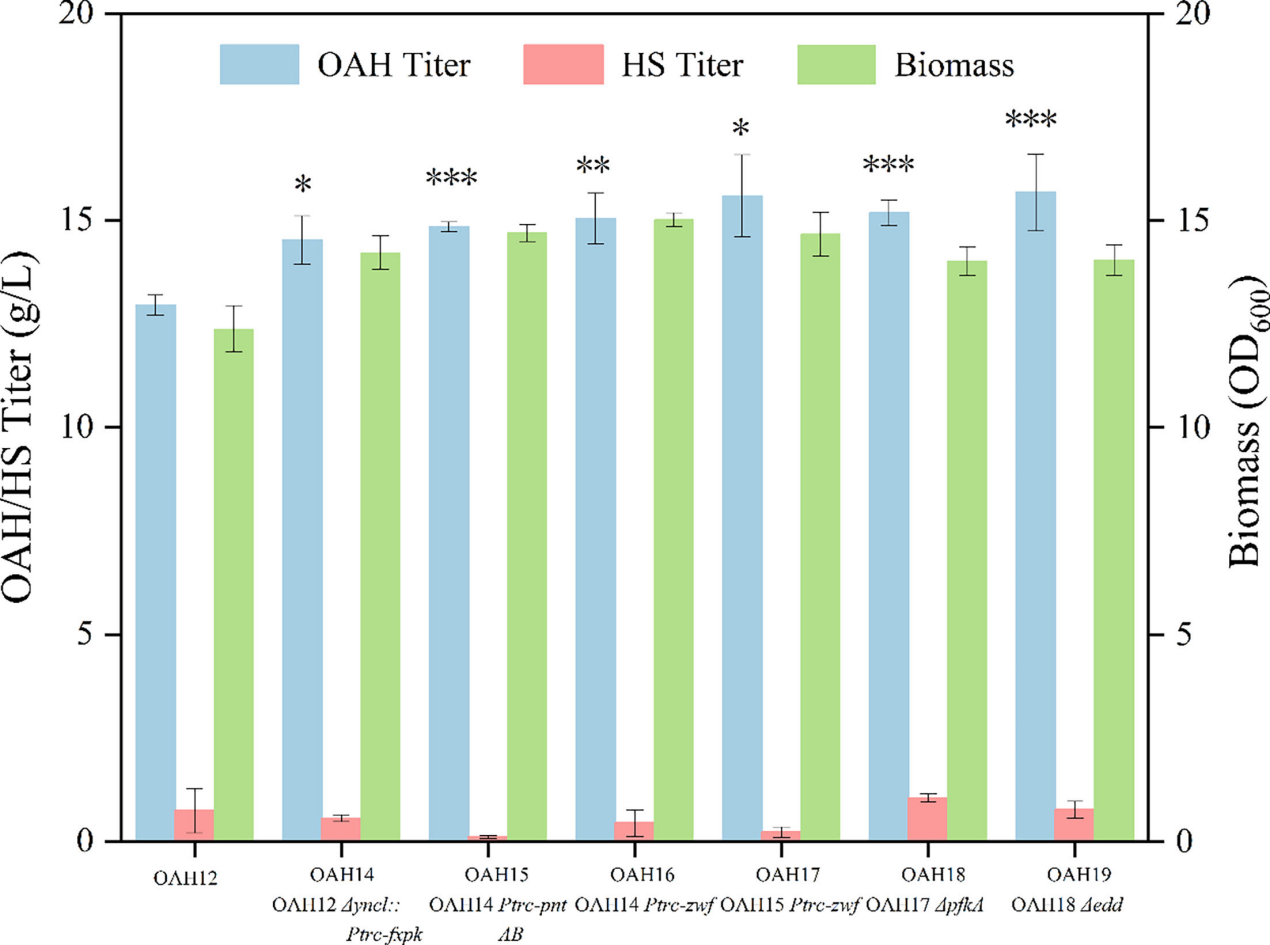
Effect of the NOG pathway construction on OAH production.

During the metabolic pathway of *E. coli*, pyruvate is catalyzed by the pyruvate dehydrogenase system to produce acetyl-CoA. However, pyruvate would lose one molecule of carbon atom during the decarboxylation process, which was a limitation for the biobased products with acetyl-CoA as a precursor [34]. NOG pathway (non-oxidative glycolysis pathway, Figure 1) decomposes 1 molecule of fructose-6-phosphate (F6P) into 3 molecules of acetyl phosphate (AcP), which can reduce carbon loss and improve carbon source utilization [35,36]. Acetyl phosphate can further synthesize acetyl-CoA by PTA, so the NOG pathway can achieve complete carbon conservation when glucose is catabolized to acetyl-CoA, which is a very economical pathway during the glucose metabolism [37]. In this study, the key enzyme phosphotransferase (encoded by gene *fxpk_ba_*) derived from *Bifidobacterium adolescentis* was overexpressed to construct the NOG pathway based on strain OAH12, and strain OAH14 was obtained. The results of shaking flask fermentation in Figure 5 showed that the OAH titer increased to 14.52 g/l, 12.12% higher than that of strain OAH12.

However, compared with the glycolysis pathway, the NOG pathway does not produce NAD(P)H, which was a key substance that transfers cellular energy and provides reduction carriers required for oxidation in intracellular biosynthesis and decomposition reactions [38,39]. During the OAH synthetic pathway, the production of one molecule of l-homoserine consumes two molecules of NADPH. Therefore, the coenzyme cycle pathway should be strengthened on the basis of the NOG pathway constructed above. Previous studies have shown that overexpression of pyridine nucleotide transhydrogenase (encoded by gene *pntAB*) can effectively promote the NADPH regeneration, and this strategy has been successfully applied to the modification of amino acid metabolic pathways, such as l-lysine and l-homoserine, significantly increasing the output of target metabolites [24,40]. In addition, glucose-6-phosphate (G6P) could be catalyzed by the 6-glucose phosphate dehydrogenase (encoded by the gene *zwf*) in the first step of the oxidation stage during the pentose phosphate pathway, and NADPH regeneration was accompanied by this pathway [41]. Therefore, in order to enhance NADPH recovery and regeneration, the genes of *pntAB* and *zwf* were strengthened with strong promoter trc separately and jointly in the genome of strain OAH14, and the strains of OAH15, OAH16 and OAH17 were obtained, respectively. The results of shaking flask fermentation and intracellular NADPH concentration were shown in Figure 5 and Supplementary Figure S2, respectively. It indicated that the strengthening of *pntAB* and *zwf* jointly could improve the NADPH generation and further increase the OAH production to 15.59 g/l, 7.37% higher than that of strain OAH14.

From the above results, it could be concluded that the NOG pathway played a crucial role in the biosynthesis of OAH; therefore, subsequent studies focused on optimizing the key nodes within this pathway. In the glycolytic pathway, fructose-6-phosphate (F6P) could be catalyzed to fructose-1, 6-diphosphate (FBP) by the 6-phosphofructokinase I (PFK-I, encoded by gene *pfkA*) [42]. In the Entner-Doudoroff (ED) pathway, glucose-6-phosphate (G6P) could be converted to 2-dehydro-3-deoxy-6-phosphogluconic acid (KDPG) by phosphogluconate dehydratase (encoded by gene *edd*) [43]. Therefore, the genes of *pfkA* and *edd* were knocked out successively, constructing strain OAH18 and OAH19. As a result, OAH production was not greatly improved (Figure 5). It was hypothesized that the introduction of the NOG pathway contributed to enhancing carbon source utilization. In contrast, the attenuation of the EMP and ED pathways exhibited minimal impact on carbon source utilization. The effect of the NOG pathway on carbon source utilization may not have been prominently reflected in shaking flask fermentation. Therefore, strain OAH19 was fermented in a 5 l bioreactor. The results in Supplementary Figure S3 showed that the OAH titer reached 53.98 g/l, and the yield was 0.35 g/g glucose. However, the fermentation period of this strain is still longer than other reported strains.

### Improvement of glucose uptake to regulate the cell growth

**Figure 6:**
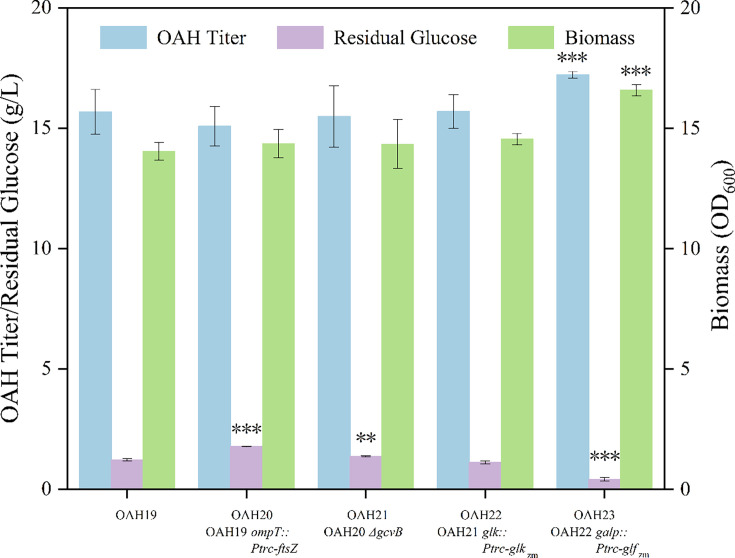
Effect of modification of cell division and glucose transport system on OAH production.

The fermentation process in the 5 l bioreactor showed that the strain OAH19 exhibited slower growth rate and longer fermentation period, which would adversely affect the production of OAH. It was reported that engineered cell division, including enhanced cell growth and cell volume, can change production characteristics during industrial microbial fermentation [44]. The down-regulation of the gene *ftsZ* could prevent proper fission assembly and thus the high-density culture of bacteria, while overexpression of *ftsZ* can effectively shorten the cell division cycle [44]. GcvB is a trans-coding regulatory sRNA in gram-negative bacteria, such as *E. coli* and *Salmonella* [45]*,* which works synergistically with the chaperone protein Hfq to produce regulatory effects on target genes. GcvB regulates relevant genes involved in amino acid metabolism through the post-transcriptional level. By knocking out the gene *gcvB*, the inhibition of amino acid transport could be removed. Therefore, in order to investigate the correlation between OAH synthesis and cell division, P_*trc*_ *-ftsZ* was introduced into the *ompT* gene site of strain OAH19, and strain OAH20 was constructed. Meanwhile, the gene *gcvB* was knocked out on strain OAH20 to gain strain OAH21. The results in Figure 6 showed that after the modification of the cell division, OAH production and cell growth did not change significantly. It might be due to the experiments being conducted in the shaking flask, which would not reflect the growth curve of the cells. In addition, it was found that the residual glucose concentrations in the fermentation broth of strain OAH20 and strain OAH21 were still more than 1 g/l, which indicates that the glucose uptake capacity of the strains still needs to be improved.

In *E. coli*, there is a glucose transport system composed of galactose transporter (GalP) and glucokinase (Glk) in addition to the PTS system [46]. These two proteins are encoded by the genes of *galp* and *glk*, respectively. In this glucose transport pathway, 2 mol ATP is consumed for transporting 1 mol glucose [45,47], while Glf protein from *Zymomonas mobilis* (encoded by gene *glf_zm_*) can transport glucose through co-ordinated diffusion. Under normal circumstances, the concentration of glucose in the fermentation medium is high, so the application of Glf instead of GalP in engineered bacteria is more conducive to improving the utilization capacity of glucose [48]. It was reported that the Glf-Glk system derived from *Zymomonas mobilis* had higher efficiency than the natural system of *E. coli* [49]*.* In order to improve the uptake of glucose, the strains of OAH22 and OAH23 were constructed by replacing the gene *glk* with *glk*_*zm*_ (from *Zymomonas mobilis*) and galP with *glf*_*zm*_, respectively. The results of shaking flask fermentation in Figure 6 indicated that the final OAH titer of the strain OAH23 reached 17.23 g/l, 11.23% higher than that of strain OAH21. The function of Glf is analogous to that of GalP. However, while GalP operates as a H^+^ symporter and requires energy for glucose transport, Glf functions without the need for additional energy input. Consequently, Glf exhibits superior energy efficiency in this metabolic process compared with GalP.

### Fed-batch fermentation in a 5 l bioreactor

**Figure 7:**
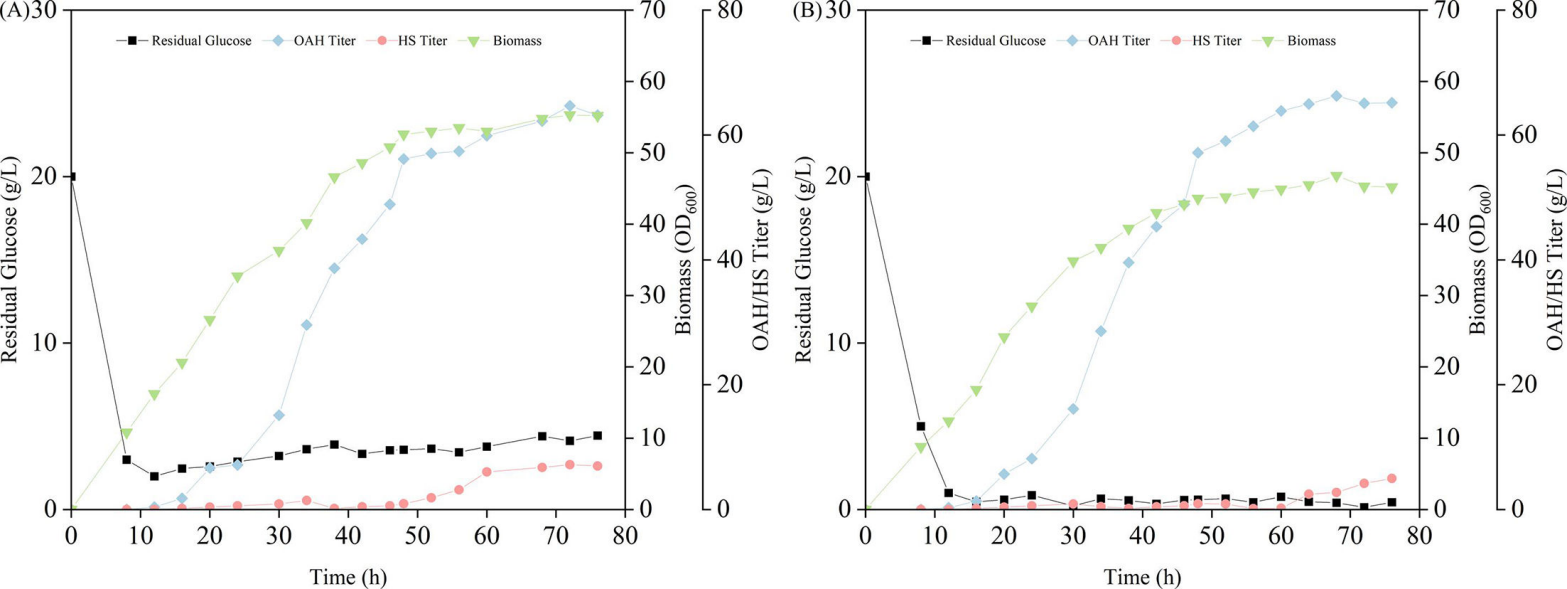
Fed-batch fermentation profiles of strain OAH23 in a 5 l fermenter. (**A**) pH-stat fed-batch fermentation; (**B**) DO-stat fed-batch fermentation

Strain OAH23 was cultured in a 5 l bioreactor using pH-stat and DO-stat feeding strategies for fed-batch fermentation, respectively, and the fermentation results were shown in Figure 7. Under the pH-stat strategy (Figure 7A), the feeding was automatically started when the pH was above 6.82. At the same time, the stirring speed is coupled with dissolved oxygen to keep the dissolved oxygen in the fermentation solution at about 10%. After fermentation for 72 h, the OAH titer reached 64.67 g/l with the yield of 0.37 g/g glucose and productivity of 0.90 g/l/h. Under the DO-stat feeding strategy (Figure 7B), the feeding is automatically started when the DO is higher than 15%, and the DO is maintained at about 10% by adjusting the stirring speed. After fermentation for 68 h, the OAH titer reached 66.25 g/l with the yield of 0.41 g/g glucose and productivity of 0.97 g/l/h. The results indicated that the DO-stat feeding strategy was more suitable for the fermentation of strain OAH23. However, the fermentation process in the 5 l bioreactor needs to be further optimized in future studies.

## Discussion

O-Acetyl-l-homoserine (OAH), a derivative of l-homoserine, relies on the supply of upstream precursors (l-aspartic acid, l-homoserine, and acetyl-CoA) and efficient glucose utilization for its biosynthesis. To achieve high-yield OAH production, this study focused on engineering three core modules in the OAH biosynthetic pathway: the l-aspartate/l-homoserine biosynthesis module, the acetyl-CoA supply module, and the glucose transport module. Corresponding targeted modification strategies were implemented, and their effects on OAH production were systematically analyzed as follows.

First, to enhance the supply of key precursors l-aspartic acid and l-homoserine, we integrated and optimized the expression of five functional genes (*ppc*, *aspC*, *asd*, *aspA*, and *metX_cg_*). This strategy effectively promoted precursor accumulation, laying a foundational basis for OAH biosynthesis. In contrast, attempts to balance acetyl-CoA supply by regulating the acetic acid-acetyl-CoA metabolic cycle failed to yield significant improvements. This may be attributed to the complex regulatory network of acetyl-CoA biosynthesis, which is not only affected by acetic acid accumulation but also co-ordinated by multiple factors such as pyruvate, fatty acids, and CoA supply [50–52]. Thus, acetyl-CoA availability in OAH biosynthesis is not constrained by a single linear pathway, and more systematic multi-pathway regulation is required to optimize its supply for OAH synthesis.

To reduce non-productive carbon loss and improve carbon utilization efficiency, we constructed a phosphoketonase-regulated non-oxidative glycolysis (NOG) pathway in *E. coli*. Unlike the native pyruvate dehydrogenase system that emits CO_2_, the NOG pathway enables the conversion of one molecule of glucose into three molecules of acetyl-CoA with complete carbon preservation [53]. The introduction of this pathway increased the OAH titer to 15.59 g/l, confirming that enhancing carbon conservation efficiency is a critical determinant for promoting OAH biosynthesis. This finding provides a valuable reference for metabolic engineering strategies targeting acetyl-CoA-dependent metabolites.

To further enhance glucose utilization efficiency, we optimized the glucose transport system by heterologously expressing the Glf-Glk glucose transport system derived from *Zymomonas mobilis*. Glf functions as a glucose facilitator protein that, when combined with Glk protein, promotes glucose transport through membrane diffusion [54]. Compared with the native ATP-dependent GalP-Glk system, the heterologous Glf-Glk system not only reduces energy consumption during glucose translocation but also exhibits a significantly higher transport rate. After introduction, the residual glucose concentration in the medium decreased to 0.41 g/l, and the OAH titer increased to 17.23 g/l. These results validate that optimizing the glucose transport system is an effective strategy to enhance carbon flux toward OAH biosynthesis by improving glucose utilization efficiency.

By integrating the above modification strategies, we successfully constructed a plasmid-free high-yield OAH-producing strain OAH23. Fermentation in a 5 l bioreactor achieved an OAH titer of 66.25 g/l, a yield of 0.41 g/g glucose, and a shortened fermentation cycle of 68 hours. This confirms that the synergistic regulation of precursor supply, carbon conservation, and glucose transport can effectively drive efficient OAH biosynthesis.

Despite these achievements, there remains scope for further optimization. Future studies could combine metabolomics and other omics technologies to identify additional rate-limiting nodes in OAH production. Targeted optimization of acetyl-CoA supply through multi-pathway regulation and refinement of fermentation processes is expected to further improve OAH titer. In summary, this study establishes a robust and scalable OAH production strategy, laying a solid foundation for its industrial application. Moreover, the developed metabolic engineering approaches and theoretical frameworks provide transferable insights for the biosynthesis of OAH and other acetyl-CoA-dependent acetylated amino acids in *E. coli*.

## Material and methods

### Strains and plasmids

All strains and plasmids used in this study are listed in Table 1. Plasmid construction was performed using *E. coli* DH5α as the host. The l-homoserine-producing strain HS33 constructed in our lab was used as the chassis strain for genome editing [19]. The gene *metX* from *Corynebacterium glutamicum* ATCC 13032 (named *metX_cg_*) was integrated into the *rpnD* locus of the strain HS33, resulting in the starting strain OAH1.

**Table 1:** Strains used in this study

Strains	Genotype	Source
*E. coli* DH5α	The cloning cost	This lab
HS33	*E. coli*-W3110-ΔJIB*-Δ*thrB*-Δ*metA*-Δ*lysA*-Δ*iclR*-Δ*ptsG*-Δ*galR*-Δ*lacI::*P_trc_-*rhtA*-P_trc_-*rhtA*-P_trc_-*eamA*-P_trc_-*metL*-P_trc_-*thrA*-P_trc_-*glk*-P_trc_-*gltB*	This lab [19]
OAH1	HS33-Δ*rpnD*::P_trc_-*metX_cg_*	This study
OAH2	OAH2-P_trc_-*aspC*	This study
OAH3	OAH2-P_trc_-*aspA*	This study
OAH4	OAH2-P_trc_-*ppc*	This study
OAH5	OAH4*-*P_trc_-*aspC*	This study
OAH6	OAH5-P_trc_-*aspA*	This study
OAH7	OAH6*-*P_trc_ *-asd*	This study
OAH7-1	OAH7-P_trc_-*metX*	This study
OAH7-2	OAH7-pACYC-*metX*	This study
OAH7-3	OAH7-pTrc99A-*metX*	This study
OAH8	OAH7-1-*ptsG_(complementation)_*	This study
OAH9	OAH8-Δ*ptsH*	This study
OAH10	OAH8-Δ*crr*	This study
OAH11	OAH8-Δ*ptsI*	This study
OAH12	OAH10*-*P_trc_ *-acs*	This study
OAH13	OAH10*-*Δ*pta-ackA*	This study
OAH14	OAH12-Δ*yncl*::P_trc_ *-fxpk_ba_*	This study
OAH15	OAH14-P_trc_ *-pntAB*	This study
OAH16	OAH14-P_trc_ *-zwf*	This study
OAH17	OAH15*-*P_trc_ *-zwf*	This study
OAH18	OAH17-Δ*pfkA*	This study
OAH19	OAH19*-Δedd*	This study
OAH20	OAH19*-ompT*::P_trc_-*ftsZ*	This study
OAH21	OAH20*-*Δ*gcvb*	This study
OAH22	OAH21*-glk*::P_trc_-*glk_zm_*	This study
OAH23	OAH22-*galp*::P_trc_-*glf_zm_*	This study

### Plasmids construction and gene manipulation

The plasmid for gene overexpression was constructed based on pTrc99A or pACYC. The target gene was amplified by a polymerase chain reaction and was cloned into the vector using a cloning expression system [55]. The primers used in this study are listed in Supplementary Table S1 of the Supplemental file. Gene manipulation was performed using a CRISPR-Cas9 system as described in the previous study [56].

### Cultivation in shaking flasks

Luria-Bertani (LB) medium was used for seed culture, contained (per liter): 10 g tryptone, 5 g yeast extract, and 10 g NaCl. The strains were inoculated into a 10 ml seed medium and cultivated at 37°C with 200 rpm for 10 h. The MS medium was used to evaluate the OAH production capacity of the constructed strains, contained (per liter): 40 g glucose, 16 g (NH_4_)_2_SO_4_, 4 g yeast extract, 1 g KH_2_PO_4_, 1 g MgSO_4_, 0.005 g FeSO_4_·7H_2_O, 0.005 g MnSO_4_·7H_2_O, 0.005 g ZnSO_4_, 0.05 g l-threonine, 0.02 g l-methionine, and 0.01 g l-lysine. CaCO_3_ (25 g/l) was used for regulation of pH, which was sterilized separately by autoclaving at 115°C for 30 min and added into the medium before inoculation. Streptomycin hydrochloride (50 μg/ml), kanamycin sulfate (50 μg/ml), chloramphenicol (25 μg/ml), and isopropylthio-β-D-galactoside (IPTG, 0.12 μg/ml) were added as needed. The seed cultures were transferred with 5% inoculum into a 250 ml shaking flask containing 20 ml MS medium, and then cultivated at 30°C with 180 rpm for 48 h.

### Fed-batch fermentation in the 5 l bioreactor

Fed-batch fermentation was conducted in a 5 l bioreactor with the working volume of 2 l (Baoxing Bioequipment Co., Ltd., Shanghai, China). The initial fermentation medium in the bioreactor contained (per liter): 20 g glucose, 17 g (NH_4_)_2_SO_4_, 4 g yeast extract, 1 g KH_2_PO_4_, 1 g MgSO_4_, 2 g betaine, 0.005 g FeSO_4_·7H_2_O, 0.005 g MnSO_4_·7H_2_O, 0.005 g ZnSO_4_, 0.5 g l-threonine, 0.2 g l-methionine, and 0.1 g l-lysine. The feeding medium contained (per liter): 500 g glucose, 10 g (NH_4_)_2_SO_4_, 12.5 g KH_2_PO_4_, 2 g betaine, 4 g l-threonine, 1 g l-methionine, and 0.5 g l-lysine. The seed was cultured in the flask at 37°C and 200 rpm for 10 h and then inoculated into the bioreactor with 10% inoculum. The incubation temperature was 30°C, and the pH was maintained at 6.8 by automatically adding NH_3_·H_2_O (50% vol concentration). The ventilation rate was 2 VVM. The fermentation process was controlled by pH-stat or DO-stat.

### Analytical methods

The biomass was measured by absorbance at 600 nm (OD_600_) using a spectrophotometer (Amersham Biosciences, Inc., Uppsala, Sweden). The concentrations of OAH, l-homoserine, and other byproduct amino acids in the fermentation broth were detected by high-performance liquid chromatography (HPLC, ThermoFisher Scientific Inc., Waltham, MA, U.S.A.) with online derivatization and gradient elution using C18 column (4.6 × 250 mm, 5 μm; Welchrom) at 35 °C and a detection wavelength of 338 nm [57–59]. The fermentation supernatant and the standard solution were filtered through a 0.22 μm aqueous membrane, and the contents of various organic acids in the samples were detected by high performance liquid chromatography (HPLC) on an Aminex ®HPX-87H column (300 mm × 7.8 mm, 0.25 µm). The parameters were as follows: injection volume of 20 μl, flow rate of 0.6 ml/min, column temperature of 60°C, ultraviolet detection wavelength of 200 nm and mobile phase 5 mmol/l H_2_SO_4_. Determination of residual glucose in the fermentation broth was carried out using an M-100 biosensor analyzer from Sillman Technology Ltd. The intracellular NADPH content is measured by the NADPH assay kit; for detailed procedures, please refer to the instruction manual of the Beyotime NADP^+^/NADPH Assay Kit (WST-8 method).

## Statistical analysis

All data in the figures are expressed as mean ± standard deviation (SD) from three independent biological replicates. Statistical significance was determined using two-tailed Student’s *t*-test, and *P* values are indicated with asterisks as follows: 0.01 < *P* < 0.05 (∗), *P*<0.01 (∗∗), *P*<0.001 (∗∗∗). These statistical annotations have been consistently applied to all relevant figures.

## Supplementary material

online supplementary material 1.

## Data Availability

The data that support the findings of this study are available from the corresponding author upon reasonable request.

## Abbreviations

ACK: acetate kinase
ACS: acetyl-CoA synthetase
ASP: L-aspartic acid
ASP-P: aspartyl-phosphate
ASP-SA: aspartate semialdehyde
AcCoA: acetyl-CoA
AcP: acetyl phosphate
CIT: citrate
ED: Entner-Doudoroff
EMP: glycolysis pathway
FBP: fructose-1, 6-diphosphate
F6P: fructose-6-phosphate
FUM: fumaric acid
G3P: glyceraldehyde-3-phosphate
G6P: glucose-6-phosphate
GalP: galactose transporter
Glk: glucokinase
HS: L-homoserine
KDPG: 2-dehydro-3-deoxy-6-phosphogluconic acid
L-Lys: L-lysine
L-Met: L-methionine
L-Thr: L-threonine
MetX: homoserine acetyltransferase
MetY: acetylhomoserine sulfhydrylase
NOG: non-oxidative glycolysis
OAA: oxaloacetic acid
OAH: O-acetyl-L-homoserine
OSH: O-succinyl-L-homoserine
PEP: phosphoenolpyruvate
6 PGL: 6-phospho-D-gluconate
PPP: pentose phosphate pathway
PTA: phosphate acetyltransferase
PTS: phosphotransferase system
Pyr: pyruvic acid
TCA: tricarboxylic acid
