# Supplementary material for: Construction and regulation of microbial cell factories for enhancing the biosynthesis of O-acetyl-l-homoserine in Escherichia coli W3110
Source: Biochem J. 2026 Feb 2;483(2):161–75. doi: 10.1042/BCJ20243022 (PMC12905496; doi:10.1042/BCJ20243022)
Supplement: online supplementary material 1. [file bcj-483-2-BCJ20243022-s001.docx]

**Supplemental file**

**Title:**

Construction and regulation of microbial cell factories for enhancing the biosynthesis of O-acetyl-L-homoserine in *Escherichia coli* W3110

**Running Head:**

Biosynthesis of O-acetyl-L-homoserine in *E. coli*

**Author names and affiliations:**

Kun Niu^1^, Yi-Fan Zhao^1^, Zi-Xuan Zhang^1^, Yao-Yao Wang^1^, Kai-Di Xiang^1^, Sen Cui^1^, Zhi-Qiang Liu^1*^ and Yu-Guo Zheng^1^.

1 Key Laboratory of Bioorganic Synthesis of Zhejiang Province, College of Biotechnology and Bioengineering, Zhejiang University of Technology, Hangzhou 310014, P. R. China

***Corresponding author：**

Zhi-Qiang Liu

E-mail: microliu@zjut.edu.cn

Tel: +86-571-88320379

Fax: +86-571-88320630

**Figure caption**

**Figure S1 Regulation of the genes of *pta-ackA* and *acs* on pyruvate production.**

**Figure S2 Concentration of NADPH in different strains.**

**Figure S3 Fed-batch fermentation of strain OAH19 in the 5-L bioreactor.**

**Figure S1**

**Figure S2**

**Figure S3**

**Table caption**

**Table S1 Primers used in this study.**

**Table S1**

| Primers | Sequence (5’ to 3’) |
| --- | --- |
| p-metx-up | TTTCACACAGGAAACAGACCATGCCCACCCTCGCGCCTTC |
| p-metx-down | CCGCCAAAACAGCCAAGCTTTTAGATGTAGAACTCGATGTAG |
| pACYC-vF | ATTCGATGGTGTCCGGGATCT |
| pACYC-vR | CGTTTCACTTCTGAGTTCGGC |
| pACYC-XF | AAGCTTGGCTGTTTTGGCGG |
| pACYC-XR | GGTCTGTTTCCTGTGTGAAA |
| p-thrA-up | CATCGAGTTCTACATCTAATTTCACACAGGAAACAGACCatgCGAGTGTTGAAGTTCGG |
| p-thrA-down | CCGCCAAAACAGCCAAGCTTtcaGACTCCTAACTTCCATGAG |
| pTrc99A-XF | AAGCTTGGCTGTTTTGGCG |
| 99A-metX-XR | GCCGGATGATTAATTGTCAACAGCTCATTTCAGAATATTT |
| pTrc99A-VF | CGCCGACATCATAACGGTT |
| pTrc99A-VR | AGACCGCTTCTGCGTTCTG |
| 99A-metX-up | AAATATTCTGAAATGAGCTGTTGACAATTAATCATCCGGC |
| 99A-metX-down | CCGCCAAAACAGCCAAGCTTTTAGATGTAGAACTCGATGTAG |
| aspC-PAM-F | TAATACTAGTGCGTTTTCATCAGTAATAGTGTTTTAGAGCTAGAAATAGC |
| aspC-PAM-R | GCTCTAAAACACTATTACTGATGAAAACGCACTAGTATTATACCTAGGAC |
| aspC-P1 | CGAGTCGGTGCTTTTTTTGAATTCTCTAGATTGGCAACGGTAAAAAAGCT |
| aspC-P2 | TATCTCCTTTGTGACCACACATTATACGAGCCGGATGATTAATTGTCAAGTTTTTGCAAGACGTGAGAT |
| aspC-P3 | ACAATTAATCATCCGGCTCGTATAATGTGTGGTCACAAAGGAGATATACATGTTTGAGAACATTACCGC |
| aspC-P4 | TAGATCTAAGCTTCTGCAGGTCGACTTCAGGCTGTTAATCAGTGC |
| ppC-PAM-up | TAATACTAGTTTGACGTCACCGCTTTTACGGTTTTAGAGCTAGAAATAGC |
| ppC-PAM-down | GCTCTAAAACCGTAAAAGCGGTGACGTCAAACTAGTATTATACCTAGGAC |
| ppc-p1-up | ATTCTCTAGAGTCGACCTGCCCACCCGCGAACTGATAAC |
| ppc-p2-down | AATTCCACACATTATACGAGCCGGATGATTAATTGTCAAAACGAATAAATAGCAGGAAT |
| ppC-p3-up | CTCGTATAATGTGTGGAATTTTTCACACAGGAAACAGACCATGAACGAACAATATTCCG |
| ppc-p4-down | GGGTAATAGATCTAAGCTTCGCGATATCTTTGTTATCGAG |
| ppc-VF | CAGGCTCATCAACCTGATG |
| ppc-VR | CATCGGTATGCCATGACTG |
| metX-p1-up | GCGCCGCCATGAGGAAACCTGATTGACAATTAATCATCCGGCTCG |
| metX-P1-down | CGCCGAACTTCAACACTCGCATTTAGATGTAGAACTCGATGTAGG |
| thrA-P2-up | TACATCGAGTTCTACATCTAAATGCGAGTGTTGAAGTTCGGC |
| thrA-P2-down | GATTACGGTGCTACCTCTGACGTCAGACTCCTAACTTCCATGAG |
| flik-PAM-up | TAATACTAGTGACACCACCACATTGCCTGGGTTTTAGAGCTAGAAATAGC |
| flik-PAM-down | GCTCTAAAACCCAGGCAATGTGGTGGTGTCACTAGTATTATACCTAGGAC |
| flik-p1-up | ATTCTCTAGAGTCGACCTGCTCTCTCCAGGGGCTGGAGC |
| flik-p1-down | ATTACGGTGCTACCTCTGACGTCAGGTTTCCTCATGGCGG |
| flik-XF | CGTCAGAGGTAGCACCGTAA |
| flik-XR | TCAGGTTTCCTCATGGCGGC |
| ptsG-PAM-F | TAATACTAGTGTAAGACGTTGGGGAGACTAGTTTTAGAGCTAGAAATAGC |
| ptsG-PAM-R | GCTCTAAAACTAGTCTCCCCAACGTCTTACACTAGTATTATACCTAGGAC |
| ptsG-P1 | GAGTCGGTGCTTTTTTTGAATTCTCTAGAATCGGTTACTGGTGGAAACTG |
| ptsG-P2 | ATGCATTCTTAAACACAATTGAGAGTGCTCCTGAGTATGG |
| ptsG-P3 | GAGCACTCTCAATTGTGTTTAAGAATGCATTTGCTAACCT |
| ptsG-P4 | GATGAGTACATCCGTAACCACTAATCCGGCAGCCAGATGG |
| ptsG-P5 | CATCTGGCTGCCGGATTAGTGGTTACGGATGTACTCATCC |
| ptsG-P6 | AATAGATCTAAGCTTCTGCAGGTCGACGTGGATGGGACAGTCAGTAAAGG |
| asd-PAM-F | TAATACTAGTAAAACGCTTATGAAAAATGTGTTTTAGAGCTAGAAATAGC |
| asd-PAM-R | GCTCTAAAACACATTTTTCATAAGCGTTTTACTAGTATTATACCTAGGAC |
| asd-p1-up | CGGTGCTTTTTTTGAATTCTCTAGAACGGAATTGCCAACGGCACGATAAATGG |
| asd-p1-down | CCACACATTATACGAGCCGGATGATTAATTGTCAAATAAATGTGCCGGT |
| asd-p2-up | TTTTCACACAGGAAACAGACCATGAAAAATGTTGGTTTTATCGGC |
| asd-p2-down | GGGTAATAGATCTAAGCTTCTGCAGCCGCCGGAAGCGGCCTGGTAGGTTGCAAC |
| aspA-PAM-up | TAATACTAGTCCTCAAGCAGCATATGATCTGTTTTAGAGCTAGAAA TAGC |
| aspA-PAM-down | GCTCTAAAACAGATCATATGCTGCTTGAGGACTAGTATTATACCTA GGAC |
| aspA-P1-up | CGGTGCTTTTTTTGAATTCTCTAGAACTGTCAGATGCTTTTGCAC |
| aspA-P1-down | ACCACACATTATACGAGCCGGATGATTAATTGTCAATTTTACCCCT |
| aspA-P2-up | CGGCTCGTATAATGTGTGGTCACAAAGGAGATATACATGTCAAAC AACATTCGTATCGA |
| aspA-P2-down | GGGTAATAGATCTAAGCTTCTGCAGATCGCATCTACCAGCTTAAT |
| aspA-VF | GGCGATCGTCTTTATGATCAAGG |
| aspA-VR | CTGTTGCACCAAGGTTAACTTCCA |
| crr-PAM-up | TAATACTAGTCAACGGTGTCGATACCGAAGGTTTTAGAGCTAGAAATAGC |
| crr-PAM-down | GCTCTAAAACCTTCGGTATCGACACCGTTGACTAGTATTATACCTAGGAC |
| crr-p1-up | ATTCTCTAGAGTCGACCTGCAATTGAAATCGGCGTAATGGTGG |
| crr-p2-down | GGGTAATAGATCTAAGCTTCATAGCGGAATTTATGTCAAACCT |
| crr-p2-up | TTTACTGCTTAGGAGAAGATCTTCTTGCCGCAGTGAAAAATG |
| crr-p1-down | CATTTTTCACTGCGGCAAGAAGATCTTCTCCTAAGCAGTAAAT |
| crr-vf | CCGAAAGAAGAGAACCCGTTC |
| crr-vr | CGCCAACTTCGTGCTGTAAC |
| acs-PAM-up | TAATACTAGTTGTAGGATGTTAATAATATGGTTTTAGAGCTAGAAATAGC |
| acs-PAM-down | GCTCTAAAACCATATTATTAACATCCTACAACTAGTATTATACCTAGGAC |
| acs-p1-up | ATTCTCTAGAGTCGACCTGCTATTGATCGGGATGCTGCGG |
| acs-p2-down | AAAATTCCACACATTATACGAGCCGGATGATTAATTGTCAATGTAGGGGTATTGGCAGT |
| acs-p3-up | CGTATAATGTGTGGAATTTTTCACACAGGAAACAGACCATGAGCCAAATTCACAAACAC |
| acs-p4-dpwn | GGGTAATAGATCTAAGCTTCGAGAAGCCGCCGAAAATCAC |
| acs-VF | GTCAACACGCTCTGACTGTTC |
| acs-VR | CACACCTTCGTCGGAAGTGATC |
| pta-PAM-up | TAATACTAGTCAATGGTTCGTGAACTGTCTGTTTTAGAGCTAGAAATAGC |
| pta-PAM-down | GCTCTAAAACAGACAGTTCACGAACCATTGACTAGTATTATACCTAGGAC |
| pta-p1-up | ATTCTCTAGAGTCGACCTGCTTACCTCTACGCCCTGCCTT |
| pta-p1-down | CCACACATTATACGAGCCGGATGATTAATTGTCAACAGCGTCCAGACGACCATC |
| pta-p2-up | TTTTCACACAGGAAACAGACCGTGTCCCGTATTATTATGCTGA |
| pta-p2-down | GGGTAATAGATCTAAGCTTCATGTTGGTGTTTTTGGCACCG |
| pta-VF | CTCACCTGATCGGTATCGAAG |
| pta-VR | GCAGGTCGACTCTAGAGAATT |
| ackA-PAM-up | TAATACTAGTCATGTCGAGTAAGTTAGTACGTTTTAGAGCTAGAAATAGC |
| ackA-PAM-down | GCTCTAAAACGTACTAACTTACTCGACATGACTAGTATTATACCTAGGAC |
| ackA-P1-up | CGGTGCTTTTTTTGAATTCTCTAGAGCGATCGGCGGCATAAAACG |
| ackA-PAM-up | TAATACTAGTCATGTCGAGTAAGTTAGTACGTTTTAGAGCTAGAAATAGC |
| ackA-P1-down | GCTGAGCTGGCGGTGTGAAAGAAGTACCTATAATTGATAC |
| ackA-P2-up | GTATCAATTATAGGTACTTCTTTCACACCGCCAGCTCAGC |
| ackA-P2-down | TAGATCTAAGCTTCTGCAGGTCGACAGTGCCCTGAGACATAACGA |
| ackA-VF | GTGCCAACACCTGTCCAGAC |
| ackA-VR | AGCTTCGCCGGATCAACATT |
| zwf-PAM-up | TAATACTAGTAGAAAATTACAAGTATACCCGTTTTAGAGCTAGAAATAGC |
| zwf-PAM-down | GCTCTAAAACGGGTATACTTGTAATTTTCTACTAGTATTATACCTAGGAC |
| zwf-P1-up | CGGTGCTTTTTTTGAATTCTCTAGATTGACATTGCGATTAACATA |
| zwf-P1-down | TGTGACCACACATTATACGAGCCGGATGATTAATTGTCAAAACTGTAAACGCTTATCCAC |
| zwf-P2-up | CATCCGGCTCGTATAATGTGTGGTCACAAAGGAGATATACATGGCGGTAACGCAAACAGC |
| zwf-P2-down | TAGATCTAAGCTTCTGCAGGTCGACAAGTATTCGCCAACCTGATC |
| zwf-VF | GCCTGAGTGAGCAAGTCGAC |
| zwf-VR | TTGTCGCACACACCGAGGA |
| pntAB-PAM-up | TAATACTAGTTCGTACATGAGCAGCTTGTGGTTTTAGAGCTAGAAATAGC |
| pntAB-PAM-down | GCTCTAAAACCACAAGCTGCTCATGTACGAACTAGTATTATACCTAGGAC |
| pntAB-P1-up | CGGTGCTTTTTTTGAATTCTCTAGACCATAGCAGAAAGCAGTGCC |
| pntAB-P1-down | TGTGACCACACATTATACGAGCCGGATGATTAATTGTCAAAGCTAAATGTTACTCCGTTA |
| pntAB-P2-up | CATCCGGCTCGTATAATGTGTGGTCACAAAGGAGATATACATGCGAATTGGCATACCAAG |
| pntAB-P2-down | TAGATCTAAGCTTCTGCAGGTCGACTTTGCCGGTGGCACTTTCCC |
| pntAB-VF | CACGCCAGTTACCGCTGTTA |
| pntAB-VR | GAACGCGTCCGACATCAC |
| fxpk-PAM-up | TAATACTAGTACCCCTGATATACGACAACAGTTTTAGAGCTAGAAATAGC |
| fxpk-PAM-down | GCTCTAAAACTGTTGTCGTATATCAGGGGTACTAGTATTATACCTAGGAC |
| fxpk-p1-up | ATTCTCTAGAGTCGACCTGCAGTAGAGGGAATGGTCATTTTATA |
| pxfk-p1-down | CCACACATTATACGAGCCGGATGATTAATTGTCAACTGGCGTCCTTTTTTATCTTTTTA |
| fxpk-p2-up | TTTTCACACAGGAAACAGACCGGATCCATGACCTCGCCG |
| fxpk-p2-down | GCCTTCTTTCTTTTCCTCTTCGAATTCTCACTCGTTATCACCAG |
| fxpk-p3-up | CTGGTGATAACGAGTGAGAATTCGAAGAGGAAAAGAAAGAAGGCT |
| fxpk-p3-down | GGGTAATAGATCTAAGCTTCAATTGCAATGACGTCTTTATCATC |
| fxpk-VF | GGCAACTCTTCGGGTTAGATG |
| fxpk-VR | CTTTATTTAGCCGCCCCTGG |
| pfkA-PAM-up | TAATACTAGTTCTGACATGATCAACCGTGGGTTTTAGAGCTAGAAATAGC |
| pfkA-PAM-down | GCTCTAAAACCCACGGTTGATCATGTCAGAACTAGTATTATACCTAGGAC |
| pfkA-P1-up | ATTCTCTAGAGTCGACCTGCTACGCATGGGATATGAGGCG |
| pfkA-P1-down | TCTGCCTTTTTCCGAAATCAGACTACCTCTGAACTTTGGAAT |
| pfkA-P2-up | TCCAAAGTTCAGAGGTAGTCTGATTTCGGAAAAAGGCAGATTC |
| pfkA-P2-down | GGGTAATAGATCTAAGCTTCGTGACTGACGAATCACCACG |
| edd-PAM-up | TAATACTAGTCCTATAACGACATGCTCTCCGTTTTAGAGCTAGAAATAGC |
| edd-PAM-down | GCTCTAAAACGGAGAGCATGTCGTTATAGGACTAGTATTATACCTAGGAC |
| edd-p1-up | ATTCTCTAGAgtcgacCTGCTGGATCTGAGCTATTCAGAAAC |
| edd-p1-down | CTGATTACAAATTTGTCGTCAAAGGCTCCTGAAATTGAGTTG |
| edd-P2-up | ACTCAATTTCAGGAGCCTTTGACGACAAATTTGTAATCAGGC |
| edd-p2-down | GGGTAATAGATCTAAGCTTCTGGGAGAACGGACCCGCGAT |
| ftsZA-PAM-F | TAATACTAGTATTGCCGTTCATAATCCCTGGTTTTAGAGCTAGAAATAGC |
| ftsZA-PAM-R | GCTCTAAAACCAGGGATTATGAACGGCAATACTAGTATTATACCTAGGAC |
| ftsZA-P1-up | ATTCTCTAGAGTCGACCTGCGAGGGGGGATATAGATTTATATA |
| ftsZA-p1-up | TAAGTTCCATTGGTTCAAACATAGCAAGCTCCTTAGAGATAAAAG |
| ftsZA-P2-up | TTTTATCTCTAAGGAGCTTGCTATGTTTGAACCAATGGAACTTAC |
| ftsZA-P2-down | CTGCTGGCTGTAATGCTGTTTTGTTAATCAGCTTGCTTACGCAGG |
| ftsZA-P3-up | CTGCGTAAGCAAGCTGATTAACAAAACAGCATTACAGCCAGC |
| ftsZA-P3-down | GGGTAATAGATCTAAGCTTCACGGCGCGTCTGACGGTC |
| ftsZA-VF | GCCAGATGTTGATAACGCGTC |
| ftsZA-VR | GACTGCGTTCTGCTTCTTGC |
| gcvB-PAM-up | TAATACTAGTTTAATCACTATGGACAGACAGTTTTAGAGCTAGAAATAGC |
| gcvB-PAM-down | GCTCTAAAACTGTCTGTCCATAGTGATTAAACTAGTATTATACCTAGGAC |
| gcvB-p1-up | ATTCTCTAGAGTCGACCTGCGTCAATTCCCGGATAAGCTGAAT |
| gcvB-p1-down | GCCGCCACTATAGGTATTTGC |
| gcvB-p2-up | CAAATACCTATAGTGGCGGCTACCTTGCGATCGCGAATTAC |
| gcvB-p2-down | GGGTAATAGATCTAAGCTTCAGACGCCCGCATCCTGTAGT |
| gcvB-VF | CACATCAACATCATCCGCCAG |
| gcvB-VR | CTGTTGGGTGGCTTCAACCAC |
| galp-PAM-up | TAATACTAGTCTGACGCTAAAAAACAGGGGGTTTTAGAGCTAGAAATAGC |
| galp-PAM-down | GCTCTAAAACCCCCTGTTTTTTAGCGTCAGACTAGTATTATACCTAGGAC |
| galp-p1-up | CACCAAAAGGGCTCAATTATATCAACGTTGTTATCTCTTGTCAACACCGCCAGAGATAAGATGCCCTCCAATATGGTTA |
| galp-p1-down | TAAACTGACTGACGCACCAAAAGGGCTCAATTATATCAACGTTGTTATCTCTTGTCAAC |
| galp-p2-up | ATAATTGAGCCCTTTTGGTGCGTCAGTCAGTTTAAACCAGGAAACAGCTATGACAAAGTATGCATTAGTCGG |
| galp-p2-down | TTACAGAATGTGACCTAAGGTCTG |
| galp-p3-up | CCTTAGGTCACATTCTGTAACTCCCCAAGCTTCCTCCCA |
| galP-p3-down | GGAGCTGCACATGAACTCGAGGCGTACGGTAAGCTGATG |
| glk-PAM-up | GGAAATTGGTCATGTTTCGGGTTTTAGAGCTAGAAATAGCAAGTTAA |
| glk-PAM-down | CCGAAACATGACCAATTTCCACTAGTATTATACCTAGGACTGAGCTA |
| glk-p1-up | CTATTACCCTGTTATCCCTACGGGTCTGGTCCGGCATGAC |
| glk-p1-down | GGGCTCAATTATATCAACGTTGTTATCTCTTGTCAACACCGCCAGAGATAATTGGTGCCGCCCACATCAC |
| glk-p2-up | ACGTTGATATAATTGAGCCCTTTTGGTGCGTCAGTCAGTTTAAACCAGGAAACAGCTATGGAATTCATGAGCAGCGA |
| glf-p2-down | TTATTTCTGGCTACGCCACA |
| glf-p3-up | TGTGGCGTAGCCAGAAATAAATCCTTCCTTTTATATCGGG |
| glk-p3-down | GGAGCTGCACATGAACTCGAGCCCGCAGCGTTTTTAATTG |
